# Transcriptome predictors of coral survival and growth in a highly variable environment

**DOI:** 10.1002/ece3.2685

**Published:** 2017-05-25

**Authors:** Rachael A. Bay, Stephen R. Palumbi

**Affiliations:** ^1^ Hopkins Marine StationStanford UniversityPacific GroveCAUSA; ^2^ Present address: Institute for the Environment and SustainabilityUCLALos AngelesCAUSA

**Keywords:** climate change, coral, gene expression, reciprocal transplant, transcriptome

## Abstract

Concern over rapid environmental shifts associated with climate change has led to a search for molecular markers of environmental tolerance. Climate‐associated gene expression profiles exist for a number of systems, but have rarely been tied to fitness outcomes, especially in nonmodel organisms. We reciprocally transplanted corals between two backreef locations with more and less variable temperature regimes to disentangle effects of recent and native environment on survival and growth. Coral growth over 12 months was largely determined by local environment. Survival, however, was impacted by native environment; corals from the more variable environment had 22% higher survivorship. By contrast, corals native to the less variable environment had more variable survival. This might represent a “selective sieve” where poor survivors are filtered from the more stressful environment. We also find a potential fitness trade‐off—corals with high survival under stressful conditions grew less in the more benign environment. Transcriptome samples taken a year before transplantation were used to examine gene expression patterns that predicted transplant survival and growth. Two separate clusters of coexpressed genes were predictive of survival in the two locations. Genes from these clusters are candidate biomarkers for predicting persistence of corals under future climate change scenarios.

## 1 Introduction

As climate change affects global ecosystems, many species are faced with the need to move, acclimate, or adapt. Within many species, especially those with low dispersal, ability to thrive under climate change varies across space, because populations are often optimally adapted to local conditions (Eckert et al., 2010; Hancock et al., 2011a). Indeed, examples of adaptation to local climate can be seen in many systems, including trees (Eckert et al., 2010; Savolainen, Pyhäjärvi, & Knürr, 2007), fruit flies (Hoffmann, 2010), and humans (Hancock et al., 2011b). For example, populations of *Drosophila melanogaster* in eastern Australia exhibit opposing clines in heat and cold tolerance concordant with their respective latitudes (Hoffmann, Anderson, & Hallas, 2002). Because local adaptation actively maintains phenotypic variation, in this case climate tolerance, populations evolved to different optimal environments could be sources of beneficial standing variation important for species persistence during rapid environmental shifts.

Although the number of documented cases of local adaptation is growing (Hoffmann & Sgrò, 2011; Sanford & Kelly, 2011), disentangling evolutionary and physiological effects on different aspects of individual success is challenging, especially in nonmodel species (Merilä & Hendry, 2014). Reciprocal transplant experiments have traditionally been used to confirm local adaptation observed as higher performance of native populations (Hereford, 2009). Although theory dictates that the maintenance of this advantage in the native environment necessitates trade‐offs in other environments, measurements of single fitness traits often fail to uncover such patterns (Hereford, 2009). In some systems, trade‐offs can be explained by antagonistic pleiotropy, where single genes impact multiple fitness traits, potentially causing them to be negatively correlated. For example, in the yellow monkeyflower, *Mimulus guttatus*, individuals with increased flower size have reduced viability, and this trade‐off is associated with a small number of quantitative trait loci (Mojica, Lee, Willis, & Kelly, 2012). Alternatively, trade‐offs could be the result allocating limited energy resources, which has been shown in several vertebrate species (Gélin, Wilson, Cripps, Coulson, & Festa‐Bianchet, 2015; Koivula, Koskela, Mappes, & Oksanen, 2003).

Increasingly, genomic techniques are being employed to help define reactions to changing environments, connecting variation in gene sequence or expression to measures of performance for a more mechanistic understanding of environmental tolerance and fitness (Fournier‐Level et al., 2011; Palumbi, Barshis, Traylor‐Knowles, & Bay, 2014; Stutz, Schmerer, Coates, & Bolnick, 2015). Merging genomic techniques with classic reciprocal transplant studies allows for the identification of molecular markers that predict performance, and thus can potentially be applied to previously unstudied populations. Quantitative trait locus (QTL) mapping has been used in a number of systems, to find genomic regions that predict fitness measures in different environments (Dittmar, Oakley, Ågren, & Schemske, 2014; Hancock et al., 2011a; Lasky et al., 2015). These methods, however, are most promising for traits encoded by few loci of large effect and demand breeding studies of closely related individuals. When such conditions cannot be met, as in many wild populations, gene expression data from transcriptomic sequencing are being used to understand and predict more complex environmentally associated traits (Roop, Chang, & Brem, 2016; Rose, Seneca, & Palumbi, 2016).

Here, we used reciprocal transplantation of a reef‐building coral to investigate the impacts of acclimation and adaptation on fitness in a highly variable backreef environment. As ecosystem builders with high levels of environmental sensitivity, corals are a group of high concern under climate change scenarios (Hoegh‐Guldberg et al., 2007). An increasing number of studies, however, have shown the capacity for this group to adjust individual physiology through acclimation, and for populations to evolve via natural selection to different temperature regimes (Bay & Palumbi, 2014; Dixon et al., 2015; Howells, Berkelmans, van Oppen, Willis, & Bay, 2013). These processes are often reflected on the transcriptome level—gene expression profiles associated with increased tolerance to high temperatures have been shown in a number of coral species (Bay & Palumbi, 2015; Dixon et al., 2015; Palumbi et al., 2014; Seneca & Palumbi, 2015). We conducted a replicated transplant study of 21 individual corals moved to 12 locations, and monitored survival, growth, and gene expression. Our data show trade‐offs between high survival in stressful conditions versus high growth in more benign conditions for the tabletop coral *Acropora hyacinthus* in different thermal environments. We also show that this complex life history trade‐off is paralleled by gene expression variation in a single coexpressed gene cluster that might be developed as a biomarker for expected fitness of these corals in future conditions.

## 2 Materials and Methods

### 2.1 Multisite reciprocal transplants

Backreef lagoon pools on Ofu Island in the National Park of American Samoa provide an ideal natural laboratory for studying thermal tolerance, as adjacent pools have drastically different temperature regimes. The two pools used in this study are the highly variable (hereafter HV) pool and the moderately variable (MV) pool. The HV pool regularly exceeds 34°C during summer low tides, while the MV pool rarely reaches temperatures higher than 32°C (Craig, Birkeland, & Belliveau, 2001). In addition to variation in temperature, these pools are also known to differ in other environmental factors such as flow velocity and nutrient concentration (Smith, Wirshing, Baker, & Birkeland, 2008). Overall, the HV pool is a more dynamic environment. In August 2012, we conducted a reciprocal transplant of 21 *Acropora hyacinthus* colonies from the backreef pools in Ofu. Of these 21 colonies, 13 originated in the MV pool and eight originated from the HV pool. Twelve fragments were removed from each colony and attached with marine epoxy to plastic bolts. From these, 12 identical transplant grids were created by attaching bolts to plastic egg crate. Each transplant grid (termed “crates” from here on) contained one fragment from each parent colony. Six crates were placed at randomly chosen locations in both the HV and MV pools. Hereafter, the pool from which the coral colony was taken will be referred to as the “origin” while the pool the fragment was transplanted into will be referred to as the “location.” A HOBO temperature logger placed on each crate measured the temperature every 10 min.

### 2.2 Growth and survival

Buoyant weight for each fragment was measured at time of transplantation. In August 2013, 1 year after transplantation, we measured survival and growth for each transplanted coral fragment. Growth for living corals was measured both by counting the number of branches present and by measuring relative increase in buoyant weight after a year of growth. For both survival and growth, we used generalized linear mixed models (GLMMs) implemented in the lme4 and lmerTest packages in R to determine the impacts of origin and location. Parent colony from which the fragment was taken and crate replicate were incorporated as random factors, and the best model was determined by stepwise selection, using AIC to compare nested models. Because survival is binary, it was modeled using a binomial distribution. Reaction norms were constructed based on means and confidence intervals from the full model.

Because branches that did not survive do not have growth measurements, we are unable to incorporate survival and growth into a single model. However, as corals are clonal and therefore allow multiple experiments for a single genotype, we can nevertheless calculate survival and growth scores for each coral colony. Our data show large variances in growth and survival among transplant crates within and between pools, so we normalized both survivorship and growth for each branch by the effect of crate replicate within each pool by taking the residuals from a linear model with crate as a fixed effect. Taking the mean of these values for each colony provided a single survival score and a single growth score for each individual in each location. To test for correlations between growth and survival within and between pools, we correlated the mean crate‐normalized survival and growth scores for each colony at each location, including origin as a fixed factor. All statistical analysis was conducted in R, and scripts can be found at https://github.com/rachaelbay/Acropora-hyacinthus-transplant-experiment.

In addition to testing for differences in group means, we also examined variability in survival across individual corals, measured as standard deviation in proportional survival (not crate normalized) of all colonies from one pool of origin. To compare this measure to a null expectation, we randomized survival within pools and recalculated standard deviation 10,000 times. Comparing the observed standard deviation to the randomizations allowed us to determine whether survival variability was greater than expected by chance due to sampling error, indicating fixed differences between individual coral colonies.

### 2.3 Gene expression network analysis

All 21 individuals used for transplant experiments had been sampled a year before transplants were created and sequenced for a transcriptome‐wide population genomics study (Bay & Palumbi, 2014). Here, we utilize transcriptome‐wide gene expression from this same dataset to examine functional genomic predictors of transplant survival and growth. Briefly, we sampled one branch from each individual, extracted total RNA using the RNAqueous 4PCR kit, constructed cDNA libraries using the Illumina TruSeq mRNA kit, and sequenced individually barcoded pooled libraries on an Illumina HiSeq 2000. These sequences were quality filtered using the fastx toolkit (Q > 20, length >20 bp; http://hannonlab.cshl.edu/fastx_toolkit/) and aligned to reference transcriptome assembled by Barshis et al. (2013) using the BWA aln algorithm [see Bay and Palumbi (2014) for detailed methods], with 450,121–1.79 million reads mapping to the coral transcriptome per colony. The number of reads that mapped to each contig was counted using custom python scripts. We discarded low‐coverage contigs, which we defined as contigs with a mean of less than one read across all individuals.

Raw read counts across individuals were normalized in DESeq2. We then conducted a weighted gene coexpression analysis using the R package WGCNA (Langfelder & Horvath, 2008). We created an unsigned network topology using a merge cutoff of 0.3 and a soft‐thresholding power of 4 (Figure S4). The coexpression analysis resulted in clusters of similarly expressed genes whose overall expression is represented by the first principal component (ME value in WGCNA) of expression across all genes in that cluster. This metric of cluster expression was then tested as a predictor for normalized transplant survival and growth in both the HV pool and the MV pool for all clusters identified in the WGCNA analysis using the lm function in R. We also tested whether proportion of the thermally tolerant clade D *Symbiodinium*, as measured in Bay and Palumbi (2014), better explained survival and growth than cluster expression. For clusters with significant associations with transplant parameters, Uniprot accessions were used to investigate functional enrichment for Gene Ontology (GO) terms using DAVID v6.7 (Dennis et al., 2003) with parameters COUNT = 5 and EASE = 0.05 and a background dataset of all genes that passed our minimum‐coverage filter.

Our samples represent transcription of the parent colony a full year before transplantation. Predictive gene expression patterns would need to be stable over time and robust to environmental shifts that corals are likely to experience at this site. To test the stability of gene expression clusters associated with survival and growth in our experiment, we examined the expression of these genes in another reciprocal transplant using many of the same individuals at the same site (Seneca & Palumbi, 2015). These samples, used as controls in another experiment, were taken after 17 months of transplantation and held for 5 or 20 hr at 29°C before preserving RNA. We were therefore able to compare gene expression from our samples to four separate treatments from that experiment: corals transplanted to two locations (HV and MV pools) and at two time points (5 and 20 hr). We extracted the normalized gene expression counts for each gene in our cluster and calculated the first principle component. This measure of cluster expression was compared to the cluster expression in our experiment using a standard linear model. This analysis allows us to confirm that colony expression is stable over time and multiple environmental treatments and therefore not impacted by natural environmental fluctuations, and also that expression is stable across multiple fragments within a colony and therefore not the result of somatic mutation.

## 3 Results

### 3.1 Overall survival, growth, and temperature

In total, 252 coral fragments were transplanted across the HV and MV pools. Of these, 34 disappeared during the course of the experiment, likely dislodged by waves or carried away by damselfish. Of the remaining 218 branches, 70 (32.1%) did not survive a full year (full growth and survival results in Table S1). The remaining 148 fragments were measured for growth using both buoyant weight and branch count, which were highly correlated (*p* < .001, *R* ^2^ = .64). Because our two measures of growth were so tightly correlated (Figure S1), we use only buoyant weight in downstream analysis.

**Figure 1:**
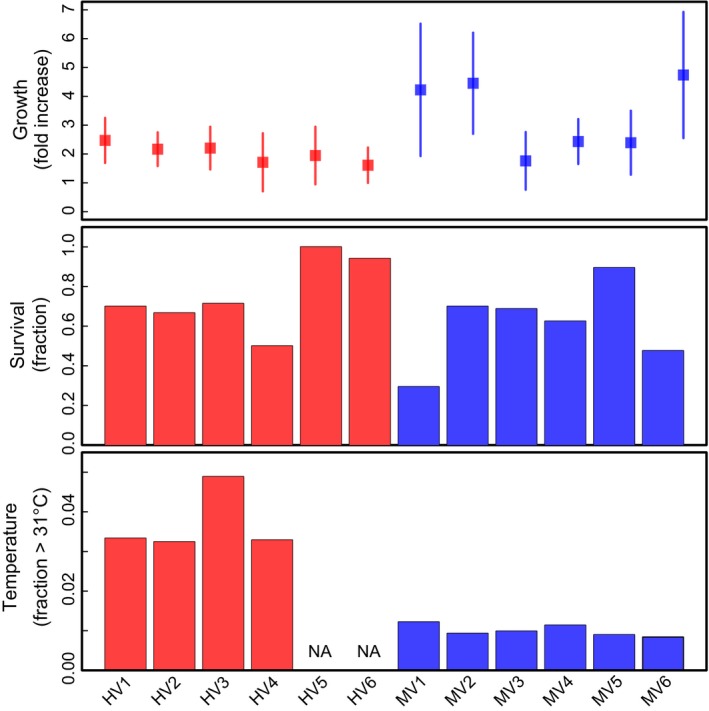
Growth, survival, and temperature for each of 12 transplant crates in the highly variable (HV) and moderately variable (MV) pools. Growth is shown as mean value of percent increase in buoyant weight across individuals, and error bars represent standard deviation

The average growth of fragments that survived was 256%, but there was significant variation between and within pools (Figure 1). For example, transplants on the crate that had the fastest coral growth grew 473%, compared to 161% for the crate with the slowest growth. Survival varied from 29% to 100% across crates. We found no correlation between growth and survival across the 12 crates when location was included in the linear model.

Ten of twelve transplant locations maintained their temperature loggers (loggers at locations HV5 and HV6 were lost). During the summer of 2012, there were 50 days when temperature reached 31°C for at least one transplant location. The highest temperatures were at the HV locations—these transplants saw temperatures above 31°C for 108–162 hr (average of 121.9 hr; 3.6% of time) from October 2012 to February 2013. By contrast, the locations in the MV pool experienced greater than 31°C for only 27–40 hr (average 31.73; 0.96% of time). The coolest locations (MV2 and MV6) had some of the highest growth rates across our study, but this pattern is not seen at location MV1, which also had a high growth rate but was one of the warmest locations in the MV pool (Figure 1). Overall, small differences in temperature among crates in the MV pool did not by themselves explain significant variation in growth or survival.

### 3.2 Effects of origin and location

**Figure 2:**
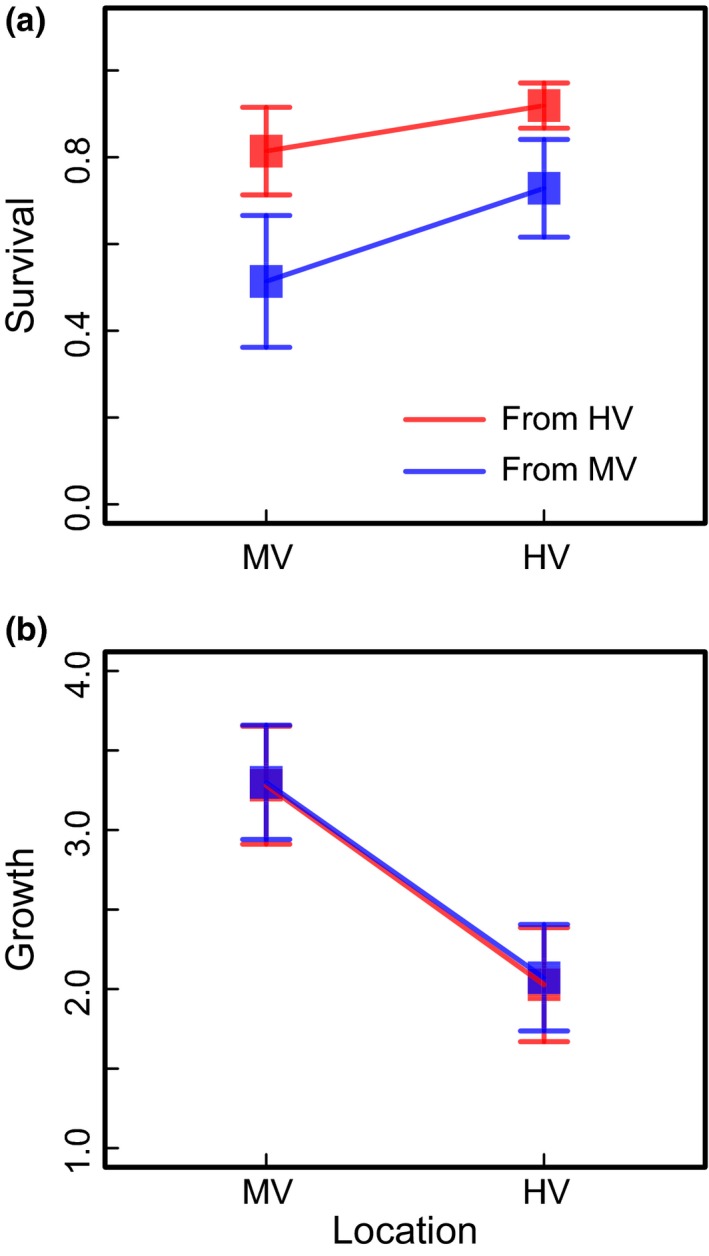
Reaction norms showing origin and location effects from reciprocal transplant of *Acropora hyacinthus* fragments between HV and MV pools. (a) Growth, shown here as percent increase in buoyant weight, is determined by location (GLMM *p *= .047). (b) Survival is significantly affected by pool of origin (GLMM *p* = .013). Here, growth and survival measures are normalized by average value for each replicate transplant crate. Error bars represent 95% confidence intervals

In spite of the large variation among transplant locations, colonies from the HV pool survived transplantation better overall (81%) compared to corals from the MV pool (58%, *p* = .013). This pattern was consistent regardless of transplant location. Corals from the HV pool had mean survival of 86% and 76% in the HV and MV pools, respectively, compared to 64% and 50% survival of corals from the MV pool (Figure 2). Mean survival was higher in the HV pool, but the effect was not significant (*p *= .17).

Growth of transplant survivors was not significantly affected by the pool of origin, but instead was determined by location (*p *= .047; Figure 2). Eighteen of nineteen coral colonies grew better in the MV pool than in the HV pool (Figure S2, Chi‐square test, *p *< .0001; colony AH82 had zero survivorship in all transplants, and AH40 survived only in the HV pool). Fragments from the HV and MV pools grew an average of 199% and 205% in the HV pool compared to 315% and 326% in the MV pool. Pool of origin had no significant effect on growth (*p *= .301).

### 3.3 Intercolony variation in survival

**Figure 3:**
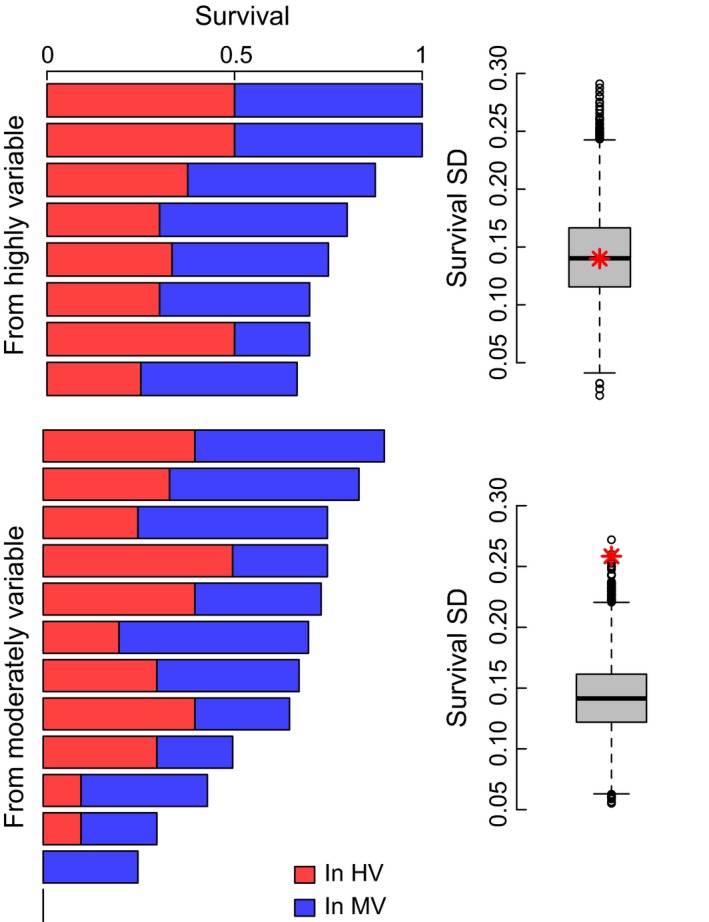
Variance in survival ability for *Acropora hyacinthus* originating from the highly variable (top) and moderately variable (bottom) pools of Ofu, American Samoa. Bar plot shows survival of transplants into the HV pool (red) and the MV pool (blue), where each bar is an individual colony. Boxplot shows the distribution of standard deviation of survival for corals from a given pool when survival is randomized across all samples. Red asterisk is the observed standard deviation of survival

In addition to nearly 40% higher survival among corals from the HV pool, these colonies also had a much lower variability in survival (standard deviation 0.14) compared to corals from the MV pool (0.26 for MV corals; Figure 3). The boxplots in Figure 3 show the observed variability in survival among individuals compared to permutations. Individuals from the MV pool have more variable survival than expected based on random mortality (permutational *p *= .0002), with many more of them showing very low survivorship. HV corals not only survive more often, but individuals tend to have uniformly high survival: Variability in survivorship across HV is not different from expected based on the mean for the HV group (permutational *p* = 0.498). We also conducted additional permutations to analyze the sensitivity of this analysis to the different sample sizes in the HV and MV pools as well as to two low‐performing individuals from the MV pool (Figure S3). In these analyses, most permutations (79% and 81%, respectively) were still significant (*p* < .05).

### 3.4 Trade‐offs in growth and survival

**Figure 4:**
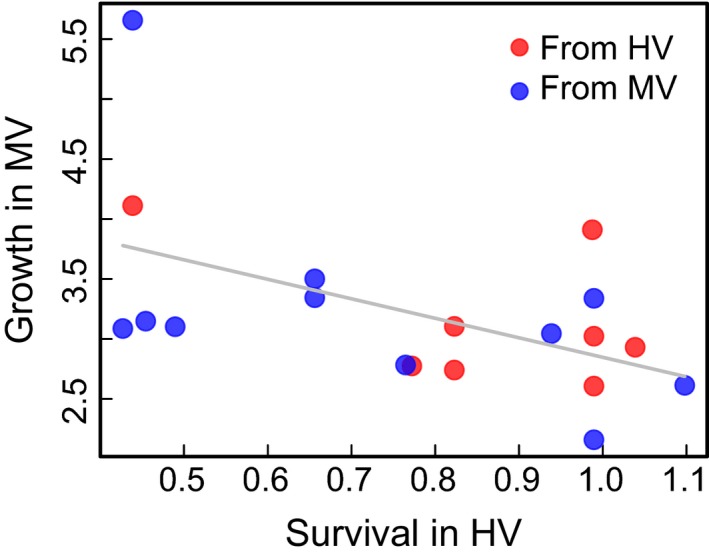
Negative correlation between survival in the highly variable (HV) pool and growth in the moderately variable (MV) pool for *A. hyacinthus* transplants (*R* ^2^ = .26, *p* = .025). Points indicate colony averages across six crates per pool, normalized for effects of crate and pool of origin

We tested for classic trade‐offs between growth and survival and found one unexpected pattern. Colonies that survived the best in the HV pool also grew poorly—but in this case, the reduction in growth was seen in the MV pool (Figure 4: *p *= .025, *R* ^2^ = .26). This analysis is somewhat sensitive to a single individual with high growth. When that individual is removed, the association is not significant (*p* = 0.08), but the trend is still negative. The same negative association exists when restricting the analysis to individuals from the MV pool: Colonies with high survival had lower growth, but in this analysis the pattern is not significant (*p *= .09), likely due to a much reduced sample size. For individuals from the HV pool, we had little power to detect a significant association, but the overall trend was also negative. We observed no relationship between survival and growth in the HV pool, possibly due to low overall growth and little variability in that location.

### 3.5 Gene expression clusters that predict transplant survival

After discarding low‐coverage contigs, we were left with 24,371 contigs for coexpression analysis. Of these, 21,680 were grouped into 27 coexpressed clusters, ranging in size from 58 to 7,718 contigs. For most clusters (19 of 27), the vast majority of variance in cluster expression could be explained by a single outlier individual, although the specific individual was not consistent across all clusters. Because variance in these 19 clusters was based on single individuals and thus unlikely to yield insight into variation across all colonies, these clusters were discarded. The remaining eight clusters were tested with standard linear models for correlations with survival and growth in both the MV and HV pools.

**Figure 5:**
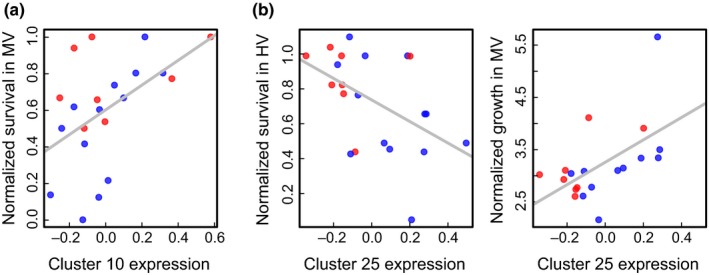
Correlations between expression of coexpressed gene clusters and survival of *Acropora hyacinthus* transplants. (a) Survival of individuals in the moderately variable (MV) pool is correlated with pretransplant expression across 403 contigs in Cluster 10 (*p *= .019, *R* ^2^ = .26). (b) Pretransplant expression of 110 contigs in Cluster 25 is negatively correlated with survival in the highly variable (HV) pool (*p *= .026, *R* ^2^ = .24) as well as growth in the MV pool (*p *= .014, *R* ^2^ = .30). Expression of coexpressed clusters is represented by the first principal component of gene expression counts across all genes in that cluster. Growth is represented by percent increase in buoyant weight

One cluster was significantly associated with survival and growth (Figure 5). Low expression in Cluster 25, a group of 110 contigs, was strongly associated with higher survival in the HV pool (*p *= .026, *R* ^2^ = .24) and lower growth in the MV pool (*p *= .014, *R* ^2^ = .30). Although this pattern was partially driven by expression differences based on the pool of origin (*t* test *p *= .006), variance within corals from the MV pool followed the same general trend of higher expression in lower surviving individuals, although it was not significant (*p *= .13). Expression in Cluster 25 also predicted growth among the subset of individuals from the MV pool (*p *= .04, *R* ^2^ = .38). These patterns parallel the trade‐off observed between growth in the MV pool and survival in the HV pool among colonies. We also tested whether gene expression or the proportion of thermally tolerant clade D *Symbiodinium* better explained fitness proxies. Based on AIC comparison, Cluster 25 expression better explained both survival in HV (expression: 1.7, symbiont: 7.5) and growth in MV (expression: 7.1, symbiont: 42.4) than did symbiont composition.

Within Cluster 25, 84 of 110 contigs had Uniprot accessions (Tables S3 & S4). DAVID analysis for enrichment of GO terms did not yield any significant categories after multiple test correction (Benjamini–Hochberg <0.1), likely due to the small number of contigs. Two biological process GO categories with significant unadjusted *p*‐values (Table 1) are both related to molecule localization. Both GO terms combined, however, only represent six contigs within that cluster.

**Table 1:** Functional enrichment of GO categories for coexpressed gene clusters correlated with survival of *Acropora hyacinthus* transplants. The table shows the ID for each GO term (term) as well as the description, number of contigs (#), percent of total contigs represented by that category (%), raw *p*‐value, and Benjamini–Hochberg corrected *p*‐value (BH)

Term	Description	#	%	*p*‐value	BH
CLUSTER 10					
GO:0032101	Regulation of response to external stimulus	6	2.61	.006	1.00
GO:0007155	Cell adhesion	16	6.96	.012	1.00
GO:0022610	Biological adhesion	16	6.96	.013	1.00
GO:0043062	Extracellular structure organization	8	3.48	.018	1.00
GO:0006355	Regulation of transcription, DNA‐dependent	22	9.57	.020	0.99
GO:0030198	Extracellular matrix organization	6	2.61	.024	0.99
GO:0051252	Regulation of RNA metabolic process	22	9.57	.026	0.99
GO:0009611	Response to wounding	9	3.91	.027	0.98
GO:0051241	Negative regulation of multicellular organismal process	5	2.17	.033	0.99
GO:0007166	Cell surface receptor‐linked signal transduction	21	9.13	.033	0.98
GO:0010557	Positive regulation of macromolecule biosynthetic process	11	4.78	.034	0.98
GO:0051094	Positive regulation of developmental process	7	3.04	.036	0.98
GO:0006790	Sulfur metabolic process	6	2.61	.037	0.97
GO:0007409	Axonogenesis	7	3.04	.042	0.98
GO:0045893	Positive regulation of transcription, DNA‐dependent	9	3.91	.043	0.97
GO:0031328	Positive regulation of cellular biosynthetic process	11	4.78	.044	0.97
GO:0045597	Positive regulation of cell differentiation	6	2.61	.045	0.96
GO:0051254	Positive regulation of RNA metabolic process	9	3.91	.049	0.96
GO:0009891	Positive regulation of biosynthetic process	11	4.78	.049	0.96
CLUSTER 25					
GO:0034613	Cellular protein localization	6	8	.027	1.00
GO:0070727	Cellular macromolecule localization	6	8	.029	1.00

Cluster 10 is correlated with survival in the moderately variable (MV) pool while Cluster 25 is correlated with survival in the highly variable (HV) pool and growth in the MV pool. Enrichment analysis was conducted using DAVID. Only biological process (BP) GO terms are shown.

A second coexpressed gene cluster was associated with survival but not growth. Higher expression in Cluster 10 (403 contigs) was associated with higher survival of transplants in the MV pool (*p *= .019, *R* ^2^ = .26). This overall pattern was true among all individuals, but was even stronger among individuals from the MV pool (*p *= .012, *R* ^2^ = .45). Cluster 10 showed neither a signal of origin (*t* test *p *= .68) nor any association with growth. Cluster 10, which was predictive of higher survival in the MV pool, was associated with 19 different GO terms. Multiple categories were associated with both cellular adhesion and transcriptional regulation.

### 3.6 Stability of gene expression clusters

We compared gene expression of Cluster 25 contigs, which was associated with survival in the HV pool and growth in the MV pool, with the same genes from a previous experiment (Seneca & Palumbi, 2015). Expression of Cluster 25 was highly stable during long‐term transplantation and short‐term common garden experiments. Gene expression from all treatments we examined from the previous experiment showed highly correlated expression of genes in this cluster with our data (*p* < 1e−4; Figure S5). Cluster 10 showed correlated expression with our experiment in only one of four treatments (Figure S6).

## 4 Discussion

We demonstrate that individual coral colonies from a single population have widely different abilities to survive transplantation in a highly variable reef environment. Colonies from the HV pool showed high survival when transplanted, whereas colonies from the more stable MV pool had on average much lower survival in the same locations. This variation in survival ability could be maintained by trade‐offs between different fitness traits; individuals with higher survivorship in the HV pool also have lower growth in the MV pool. These patterns of survival and growth were visible despite a strong overlaying signal of higher growth across all colonies in the MV pool and high variance in growth among replicate transplant sites. These reciprocal patterns were paralleled by variation in gene expression in colonies collected in their native environments before transplantation. One cluster of genes (Cluster 25) predicted both survival in the HV pool and growth in MV pool and was stable across previous transplant treatments. These genes are good candidates for predicting individual success under future environmental conditions.

### 4.1 Selection for “high survival” genotypes in a variable environment

Survival among individual coral colonies from the HV pool was both higher and less variable than corals from the MV pool. This could be the result of natural selection—genotypes that do not confer high survival rates might not survive in the extreme fluctuation in temperature and other environmental parameters observed in the HV pool. The overall variability in survival is therefore minimized in the HV pool. This is not a classic signal of local adaptation, where native individuals always outperform non‐native individuals (Hereford, 2009; Kawecki & Ebert, 2004). Rather, examination of survival alone would indicate directional selection exerted on the subset of the population that lives in the HV pool. Although we suspect that the primary selective pressure is likely temperature, as it is outside the normally tolerated range for corals in this region, the HV pool also shows large swings in oxygen, pH, and other environmental factors (Smith et al., 2008). Further studies testing corals at other sites with variable temperature regimes are necessary to show that this pattern is related specifically to temperature variability.

Previous studies on this same coral population have uncovered a number of other differences between individuals from the HV and MV pools, including gene expression, thermal tolerance, and sequence polymorphism (Barshis et al., 2013; Bay & Palumbi, 2014; Palumbi et al., 2014). Because there are no barriers to gene flow—the HV and MV pools are about 0.5 km apart—we can think of the individuals in the HV pool as those that passed through the “selective sieve” (Haldane, 1956) applied by the stressful conditions present at that location. Overall, our results suggest that this location harbors corals that are genetically and physiologically distinct from their neighbors in a more stable environment. Because our experiments were conducted on adult corals, however, we cannot completely disentangle adaptation from long‐term acclimation or epigenetic impacts. Future studies could make use of both crosses and epigenetic sequencing technologies to connect the observed physiological and genetic differences.

### 4.2 Trade‐offs between survival and growth

Fitness trade‐offs, where increased mean fitness in one environment leads to decreased fitness in another, have been observed in a number of systems (Savolainen et al., 2007) and are expected in conditions where fitness polymorphisms are maintained within populations (Levene, 1953). For example, studies of quantitative trait loci in plants have uncovered a number of convincing cases where single loci simultaneously increase fitness in one environment and decrease fitness in another (Anderson, Lee, Rushworth, Colautti, & Mitchell Olds, 2012; Hall, Lowry, & Willis, 2010). Ultimately, this trade‐off should act to maintain polymorphism within a population. In a previous study, we hypothesized that the highly heterogeneous environment in the backreef of Ofu led to maintenance of polymorphism at loci under selection (Bay & Palumbi, 2014). This is a form of spatial balancing selection, where fitness for a particular allele varies across a heterogeneous landscape (Levene, 1953; Richardson, Urban, Bolnick, & Skelly, 2014).

Our data highlight a trade‐off between survivorship and growth: Colonies with high transplant survivorship had generally slower growth in less stressful conditions that allow fast growth. This pattern suggests that local adaptation in this system could occur in the context of negatively correlated life history traits. Selection pressure associated with survival in the HV pool may favor genotypes that are better survivors in general (Figure 2). The colonies with high survival, however, have generally lower growth in the MV pool. This pattern exists even after accounting for broad differences between pools, suggesting that it is not an artifact of plastic differences between pools. We did not see trade‐offs in either growth or survival alone manifested as optimal performance of native corals in the pool of origin—the classic signature of local adaptation. However, the combined effects of survival and growth tell a different story—the trade‐off between survival in stressful environments and growth in a more stable environment may allow polymorphisms under strong selection to be maintained in the population.

Coral reciprocal transplants in other systems have yielded mixed evidence regarding local adaptation. Howells et al. (2013) transplanted colonies of *Acropora millepora* between the central and southern Great Barrier Reef and found crossing reaction norms, the classic signal of local adaptation, for bleaching, asynchronous reproduction, mortality, and growth (Howells et al., 2013). On a much smaller spatial scale, Kenkel, Almanza, and Matz (2015) found complex associations between performance measures and transplant site, but for several traits, including growth, protein content, and carbohydrate concentration, corals performed best at their native site (Kenkel et al., 2015). Our study, at a yet smaller spatial scale, shows an even more complex situation where we do not see “home site advantage” in any single fitness measure, but trade‐offs between traits occur. Even so, this trade‐off is not apparent in the means from the two sites and instead is found only when comparing traits across individuals. Perhaps, the high degree of gene flow in our system results leads to maintenance of polymorphism among individuals rather than more large‐scale trade‐offs between sites.

### 4.3 Coexpressed gene clusters predict transplant survival

Our analysis of transcriptome samples collected from field locations before transplantation highlighted two coexpressed gene clusters that predicted survival in the two backreef pools of Ofu. Rose et al. (2016) showed that the thousands of genes that shift expression during coral heat stress can be grouped into a small number of coexpressed gene clusters representing sets of genes with high correlations in expression from individual to individual. In their analysis, two clusters predicted bleaching. We analyzed our data similarly and found clusters that correlated significantly with survival and growth. Our Cluster 25 was most tightly linked to life history trade‐offs. Expression levels were positively associated with survival in the more thermally stressful HV pool and were also negatively associated with growth in the MV pool. Cluster 25 included a few genes previously associated with heat stress response in corals (Barshis et al., 2013; Seneca & Palumbi, 2015) including a caspase, one Rab‐ and two Ras‐related proteins, and a tumor necrosis factor receptor. Expression of the suite of genes included in Cluster 25 also showed remarkable stability across four separate long‐term transplant and short‐term common garden treatments in a previous experiment (Seneca & Palumbi, 2015). Because the relationships among individuals are maintained across time and environmental conditions, expression of genes in this cluster is more likely to be hard‐wired and therefore could be useful for predicting performance.

Regardless of the function of these genes, which might be obscured by poor linkage between coral protein function and human GO annotation, there is strong predictive power among genes in these clusters. We show that expression across over 100 genes can predict survival in the HV pool and growth in the MV pool, and expression of this cluster is stable across multiple experiments. Expression across this cluster might therefore be used to predict which coral colonies are most likely to survive future environmental conditions, although these patterns must first be verified in other populations. The search for biomarkers—genes whose expression correlates with the health of an individual—has been a common theme in molecular medicine (Kyle Strimbu, 2010) and is becoming increasingly common in ecological contexts (Traylor‐Knowles & Palumbi, 2014). The increasing amount of transcriptomic data allows for the discovery of predictive genes and gene families and possible application to conservation and management. In order to translate our findings into more general tools, future research should focus on testing candidate gene expression biomarkers in multiple species and a range environments.

## 5 Conclusions

Local adaptation is classically investigated by evaluating fitness of genotypes in native versus non‐native habitats and is generally thought to evolve via a series of fitness trade‐offs. In our data, trade‐offs are not visible for one fitness proxy (survival), but only appear when two complimentary fitness proxies (survival and growth) are jointly evaluated. Because so many different aspects of life history impinge on fitness (survival, growth, reproduction, etc.), there may be many cases, like this, in which basic trade‐offs inherent in local adaptation are seen in different parts of the life history. Our data also show a gene expression module that correlates well with the two life history components that combine to generate local adaptation. This discovery may make it possible to predict which colonies will transplant well and which will grow well in moderate conditions. It may also be possible to study the mechanism by which survival and growth are linked in this species by understanding the developmental wiring of this gene expression module.

## Conflict of interest

None declared.
