# Supplementary material for: Transcriptome predictors of coral survival and growth in a highly variable environment
Source: Ecol Evol. 2017 May 25;7(13):4794–803. doi: 10.1002/ece3.2685 (PMC5496549; doi:10.1002/ece3.2685)
Supplement: Supplementary file 1 [file ECE3-7-4794-s001.docx]

­­­

**Supplementary Figure 1.** Correlation between two measures of transplant growth: fold change in buoyant weight and branch count.

**Supplementary Figure 2.** Growth and survival of transplanted individuals. Growth is represented as average proportional increase in buoyant weight.

**Supplementary Figure 3.** Sensitivity of variance analysis to sample size and low-performing individuals. A) 8 MV individuals were randomly sampled to match the sample size of the HV pool. B) Same as A, removing AH82 and AH40, which had extremely low survivorship. In both cases, we sampled 100 sets and compared to a null distribution created by permuting survival among branches. Histrograms show the distribution of permutational p-values.

**Supplementary Figure 4.** Choice of soft thresholding power for construction of gene expression network in WGCNA. We used a power of 4, as it was the lowest value with model fit of R^2^>0.95

**Supplementary Figure 5.** Comparison of Cluster 10 expression 1 year before transplant in our study and the same genes in a previous reciprocal transplant study (Seneca and Palumbi 2015). Expression from our study is compared to corals transplanted into two pools (HV and MV) for 17 months after which they were kept in a short-term common garden at 29˚C for 5 or 20 hours. Gene expression from Seneca et al. is represented by the first principle component of all genes from Cluster 10.

**Supplementary Figure 6.** Comparison of Cluster 25 expression 1 year before transplant in our study and the same genes in a previous reciprocal transplant study (Seneca and Palumbi 2015). Expression from our study is compared to corals transplanted into two pools (HV and MV) for 17 months after which they were kept in a short-term common garden at 29˚C for 5 or 20 hours. Gene expression from Seneca et al. is represented by the first principle component of all genes from Cluster 25.
